# Integrated Digital Workflow for Single-Site Autogenous Bone Cylinder Augmentation and Immediate Restoration: A Case Report

**DOI:** 10.3390/dj14060348

**Published:** 2026-06-05

**Authors:** Jakub Kwiatek, Marta Leśna, Justyna Kaczewiak, Marek Szymaniak, Daria Bednarek-Hatlińska, Dominik Medyński

**Affiliations:** 1 Kwiatek Dental Clinic, Kordeckiego 22, 60-144 Poznań, Poland; 2 Military Specialist Healthcare Center Oral and Maxillofacial Surgery Department, ul. Szylinga 1, 61-787 Poznań, Poland

**Keywords:** digital dentistry, Digital CarroTrack technique, trephine, one-step crown concept, bone augmentation, autogenous bone graft, guided implant surgery, temporary crown, final crown

## Abstract

Background: The evolution of digital dentistry has enabled the development of more predictable and less invasive protocols for bone augmentation and prosthetic rehabilitation. This case report introduces an integrated digital workflow combining the Digital CarroTrack technique with the “One-Step Crown” concept to optimize clinical outcomes and efficiency. Case Description: A patient requiring alveolar ridge reconstruction and implant therapy was treated using the Digital CarroTrack technique. This approach utilized precise digital planning to simultaneously harvest an autogenous bone cylinder from the surgical site and reposition it for augmentation, eliminating the need for a secondary donor site. Following implant placement, a “one-step crown” protocol was implemented. A provisional restoration served as a template for soft tissue contouring, and its emergence profile was directly replicated in a pre-designed definitive crown. The bone cylinder fixation screw was retrieved during the final prosthetic delivery, ensuring a streamlined workflow. Results: In this case, the integrated digital approach facilitated accurate bone cylinder placement and implant positioning. At the 2-year follow-up, clinical and radiographic examinations confirmed excellent stability of both hard and soft tissues, with no marginal bone loss or soft tissue recession. The procedure reduced the number of clinical stages, treatment time, and patient morbidity compared to traditional methods. Conclusions: This case report suggests that the combination of the Digital CarroTrack Technique and the One-Step Crown concept may represent a promising, minimally invasive, and time-efficient approach for complex implant-prosthetic cases. Digital planning appeared to support procedural accuracy while reducing surgical invasiveness and the number of clinical stages. Further prospective studies with larger patient groups, objective volumetric measurements, longer follow-up, and dedicated cost-effectiveness analyses are needed to confirm the predictability, clinical effectiveness, and potential economic benefits of this approach.

## 1. Introduction

The first permanent molar plays a crucial role in the development of dental arches and the formation of occlusion. It defines the functional boundaries of the dental arch and provides posterior occlusal support points, determining the proper relationships within the stomatognathic system. Due to their position and anatomical structure, these teeth bear the greatest masticatory loads, playing a vital role in bite stabilization and the biomechanics of the masticatory organ [1]. Since they are the first permanent teeth to emerge in the oral cavity, their morphology, challenges in maintaining daily hygiene, and frequent hypomineralization make them the most commonly lost teeth [2]. Tooth loss in the posterior segment of the mandible affects masticatory function and the aesthetics of the patient’s smile, thereby representing a significant clinical problem [3].

Modern dentistry offers various methods for treating tooth loss, among which implants constitute the main strategy in their restoration, providing high functionality and favorable aesthetic integration with natural dentition [4]. The most frequent location for single implant placement is the posterior segment of the mandible, particularly the region of the first molar [5].

A fundamental challenge in implant surgery is the progressive atrophy of the alveolar process, manifested by bone loss in both horizontal and vertical planes. These limitations can be effectively eliminated through the application of advanced augmentation procedures. To restore the lost bone volume, various biomedical materials are utilized, including autogenous grafts, allogenic materials (allografts), xenogenic materials, and synthetic alloplastic materials [6].

Autologous transplantation represents the “gold standard” in bone defect regeneration and promotes proper implant osseointegration due to its osteogenic, osteoinductive, and osteoconductive attributes [7]. However, autogenous bone grafts harvested from the mandibular line require the procedure to be performed at two surgical sites—the donor and recipient sites—which increases the invasiveness of the procedure, heightens postoperative morbidity, and raises the technical difficulty of the operation [8].

In the “Carrot Technique” of autologous bone graft augmentation, popularized by Khoury, a cylinder-shaped material is harvested using a specialized bone trephine, thereby defining the site for implantation. Subsequently, this cylinder is stabilized using titanium osteosynthesis screws on the lateral side relative to the planned implant position [9].

Nevertheless, this method can be highly technically demanding in cases of a thin alveolar ridge or the proximity of vital anatomical structures. Adequate stabilization of the bone core may also be challenging due to the necessity of selecting the appropriate screw length and insertion angle.

The Digital CarroTrack Technique represents a proprietary, digital modification of the “Carrot Technique”. It utilizes a specially designed guiding sleeve (Figure 1 and Figure 2) dedicated to a trephine drill (patent application no. PL 449269), which ensures high precision during the harvesting of an autogenous bone cylinder and its subsequent guided placement. Unlike the conventional approach, the digital modification integrates preoperative implant planning, graft harvesting, fixation screw positioning, and prosthetic planning within a CAD/CAM workflow. This approach allows for enhanced control over the surgical procedure, particularly in the vicinity of critical anatomical structures.

The final stage of prosthetic rehabilitation was performed using the original ‘one-step crown’ protocol. This technique eliminates the need for secondary intraoral registration (i.e., rescanning or conventional impression taking). The final restoration is a direct digital replica of the temporary crown, with the critical distinction being the occlusal adjustment: while the temporary crown is kept out of occlusion, the final crown is precisely adapted to the previously registered occlusal conditions. Since the subgingival part of the crown remains unchanged, the emergence profile is fully preserved, fitting perfectly into the soft tissue architecture. This approach may help minimize soft tissue collapse, which is a common issue during conventional impression procedures, and may contribute to more predictable final aesthetic and functional outcomes.

## 2. Case Description

### Methods

To provide a structured overview of the clinical workflow, the main treatment components were summarized according to four methodological stages: Planning, Surgical Protocol, Prosthetic Protocol, and Follow-up. As some stages partially overlapped during treatment, they are presented separately in Table 1 for clarity.

A 45-year-old female patient, generally healthy, non-smoking, classified as ASA I, and not taking any regular medications, presented to Kwiatek Dental Clinic with a missing tooth in position 36. Following subjective and objective examinations, a comprehensive diagnostic workflow was initiated. This included Cone-Beam Computed Tomography (CBCT) (Orthophos SL 3D, Dentsply Sirona, Bensheim, Germany) (Figure 3 and Figure 4), intraoral scans (TRIOS, 3 Shape, Copenhagen, Denmark) (Figure 5), and clinical photography (Figure 6) to plan the implant placement at the site of the missing tooth 36.

Implant-prosthetic treatment was planned according to the Digital CarroTrack Technique and the ‘one-step crown’ protocol. The initial stage involved planning the implant position in the region of tooth 36 using Zirkonzahn.Implant-Planner (Version 5.0) software (Figure 7).

The next stage involved planning the positioning of bone graft fixation screws to increase the volume of hard tissues at the implantation site. Stabilization of the autologous graft using titanium screws ensures adequate mechanical stability, promotes graft osseointegration, and enables predictable outcomes in implant treatment [10].

Following the verification of the implant and fixation screw positions, a surgical guide was designed, as the patient presented with a single missing mandibular tooth and sufficient remaining dentition to provide stable support. Inspection windows were incorporated into the guide design to allow for intraoperative verification of complete seating along the entire supporting surface and to confirm the absence of guide mobility before trephine use. The trephine sleeve was designed with a diameter corresponding to the original MIS MGUIDE surgery sleeve, allowing for compatibility with the MIS MGUIDE surgical instrumentation and enabling exchange of the surgical screw when required. The sleeve length was adapted to the original drills from the MIS guide surgery kit. The guide incorporated master sleeves for implant placement and specialized sleeves for a trephine system developed by the authors (patent pending no. PL 449269), as well as sleeves for drilling stabilizing screw channels, based on standard pinning sleeves (Figure 8). Furthermore, the guide featured a T-shaped stabilizing system with a ring integrated at the junction of the elements, providing additional intraoperative stability via the use of a surgical suction tip (Figure 9). This approach is consistent with the understanding that computer-aided static navigation can achieve high accuracy in dental implant placement, provided that the surgical guide is appropriately selected and tailored to the patient’s specific anatomical conditions [11].

Initially, the preoperative clinical condition of the edentulous ridge at site 36 was assessed (Figure 10). Subsequently, the surgical guide underwent a clinical try-in to ensure adequate seating and stability prior to the commencement of the surgical procedure. (Figure 11).

First, the implantation site was marked using a drill through the surgical guide (Figure 12). Following this, a full-thickness flap was incised and reflected to displace the soft tissues and expose the underlying bone (Figure 13 and Figure 14) and allow for final positioning of the 3D-printed guide (Figure 15).

According to the Digital CarroTrack Technique protocol, the first stage of site preparation involved the use of a trephine inserted through the dedicated sleeve. The trephine used for harvesting the autogenous bone cylinder had an external diameter of 3.1 mm and an internal diameter of 2.1 mm, corresponding to the size of the harvested bone core. The drilling protocol was performed at 200 rpm under maximum external sterile saline irrigation delivered from the physiodispenser. The individualized trephine sleeve was designed with an extended guiding length to maximize instrument stabilization and improve control during bone cylinder harvesting. Upon completion of the preparation, the bone core was retrieved from the trephine, providing autogenous bone material for augmentation (Figure 16 and Figure 17).

The next step involved using a fixation pin drill to prepare the site for the bone graft stabilizing screw. An MIS C1 implant (3.75 × 8 mm; MIS Dental Implants) with a B+ sandblasted and acid-etched surface, platform-switching design, and internal conical connection with a 2.00 mm depth and 12° cone was then inserted into the prepared osteotomy with an insertion torque of 42 Ncm (Figure 18). Following implant placement, a digital scan was taken for the fabrication of a temporary crown (Figure 19). The implant position was transferred using a standard MIS scanbody provided by the manufacturer. Intraoral scanning was performed according to the standard TRIOS scanning strategy for a single missing mandibular tooth. Finally, the autogenous bone graft was secured with a titanium fixation screw 8 mm in length (Surgident, Korea; distributed by 3Z), ensuring precise adaptation and reconstruction of the missing bone tissue (Figure 20, Figure 21 and Figure 22).

On the day of the procedure, a temporary crown previously designed in Exocad to shape the planned emergence profile was delivered. The provisional crown was placed out of functional occlusion to support stable osseointegration and minimize the risk of micromovements (Figure 23 and Figure 24). This approach prevents the formation of fibrous tissue during the initial healing phase, providing optimal conditions for the long-term success of the implant treatment [12].

The wound was closed with Dafilon 5.0/16 sutures (B. Braun). Standard postoperative instructions were provided, including a 5-day course of antibiotic therapy, analgesics as needed, and chlorhexidine mouthwashes, and a schedule of follow-up visits was established. Two weeks post-surgery, uneventful healing was observed, and the sutures were removed.

At the 7-week follow-up, the clinical examination revealed excellent soft tissue health. Proper osseointegration was confirmed through radiographic assessment (Figure 25 and Figure 26).

Four months postoperatively, the final prosthetic phase was initiated (Figure 27 and Figure 28). Utilizing the ‘one-step crown’ technique, the definitive restoration was delivered in a single appointment. After removing the provisional crown, the fixation screw was located and atraumatically retrieved, followed by the immediate placement of the final restoration (Figure 29 and Figure 30). After removing the provisional crown, the fixation screw was located and atraumatically retrieved through the previously shaped emergence profile, followed by the immediate placement of the final restoration (Figure 29 and Figure 30). The exact screw position was identified based on photographic documentation, allowing access through an approximately 3 mm incision made with a 15C scalpel. This approach enabled screw removal without flap elevation, thereby preserving the peri-implant soft tissues and the emergence profile for the definitive crown. After clinical evaluation and acceptance of the provisional restoration, the definitive crown was designed by replicating the submucosal portion of the provisional crown. The prosthetic design was completed in Exocad, and the definitive restoration was milled using an imes-icore milling unit. Occlusion was designed according to the existing occlusal conditions and verified intraorally with articulating paper. No laboratory or intraoral occlusal adjustments were required. Due to the use of a provisional crown and the precise replication of the emergence profile, favorable gingival papilla contouring was achieved, allowing for harmonious adaptation to the definitive crown. This approach may help reduce a common complication in implant-supported molar restorations: food impaction in the interproximal spaces [13].

A follow-up CBCT was performed to compare the clinical outcome with the preoperative status (Figure 31 and Figure 32). The analysis revealed a normal radiographic appearance with complete bone remodeling in the surgical area. Furthermore, the final implant position showed high accuracy and alignment with the initial digital treatment plan created during the planning phase.

At the 2-year follow-up, clinical and radiographic examinations showed stable hard- and soft-tissue conditions (Figure 33 and Figure 34). CBCT-based assessment using the “compare” function demonstrated an increase in horizontal ridge width from 5.02 mm preoperatively to 7.31 mm postoperatively, corresponding to a horizontal bone gain of 2.29 mm (Figure 35). Soft-tissue conditions were assessed clinically using a periodontal probe. During treatment and follow-up, no complications or adverse events were observed, and no additional grafting procedures or soft-tissue interventions were required.

## 3. Discussion

The application of the Digital CarroTrack technique enabled the simultaneous harvest and deployment of autogenous bone within a single surgical site, eliminating the need for a secondary donor site access. This approach minimizes tissue trauma, promotes graft volume preservation, and reduces resorption. The integration of digital implant planning with the augmentation procedure may contribute to improved precision in bone cylinder harvesting and placement, potentially enhancing the accuracy and predictability of the surgical procedure. This is consistent with previous evidence indicating that static computer-assisted implant surgery can provide clinically acceptable accuracy in most clinical situations; however, the final accuracy may vary depending on patient-related and guide-related factors, including the type of guide support and the extent of edentulism [14].

In the conventional “Carrot Technique” of autogenous bone graft augmentation, popularized by Khoury, a cylindrical bone graft is harvested with a trephine and subsequently stabilized on the lateral aspect of the planned implant site using titanium osteosynthesis screws [9]. Although clinically effective, this approach may be technically demanding in narrow ridges or in close proximity to vital anatomical structures. In particular, achieving stable fixation of the bone cylinder requires careful selection of screw length and insertion angle. In this context, the Digital CarroTrack technique may offer a practical digital modification of the original concept by improving guidance during graft harvesting and positioning, while potentially reducing technical variability.

Autogenous block grafts remain a well-established option for ridge augmentation, as they allow for predictable horizontal bone gain. However, their success depends on rigid stabilization, close adaptation to the recipient site, and adequate revascularization during healing. These procedures may also be associated with donor-site morbidity, graft resorption, and increased surgical complexity [10,15]. In this context, the Digital CarroTrack technique may offer a less invasive alternative by enabling harvesting and repositioning of autogenous bone within the same surgical site, while reducing the need for a secondary donor area.

Ridge splitting is another established approach for horizontal ridge augmentation, particularly in narrow ridges where buccal and lingual cortical plates are separated by cancellous bone. However, this technique may require extensive flap elevation, corticotomies, controlled expansion of the bone segment, and in some cases a two-stage surgical protocol to reduce the risk of unfavorable fractures and preserve vascularity. Compared with such procedures, the Digital CarroTrack technique may represent a less traumatic alternative, as it enables guided harvesting and repositioning of autogenous bone within a single surgical intervention. This may reduce surgical morbidity, shorten treatment time, and avoid the need for a second-stage ridge expansion procedure.

The ‘one-step crown’ concept achieved predictable aesthetic and functional outcomes by utilizing the provisional restoration as a template for soft tissue contouring. Eliminating the need for secondary intraoral impressions and the direct replication of the emergence profile into the definitive restoration simplified the prosthetic workflow and shortened treatment time.

Furthermore, the ability to retrieve the bone cylinder fixation screw during a single appointment, while simultaneously delivering the pre-designed final crown, maintained the stability of the contoured soft tissues.

## 4. Conclusions

This case report suggests that careful technique selection and modification, supported by digital planning, may improve procedural accuracy while reducing invasiveness and the number of clinical stages. Within the limitations of a single case, the Digital CarroTrack technique appeared to support efficient autogenous bone harvesting and repositioning within one surgical site. However, further prospective clinical studies with larger patient groups, objective volumetric measurements, and longer follow-up are required to confirm the predictability, clinical effectiveness, and potential economic benefits of this approach.

## Figures and Tables

**Figure 1:**
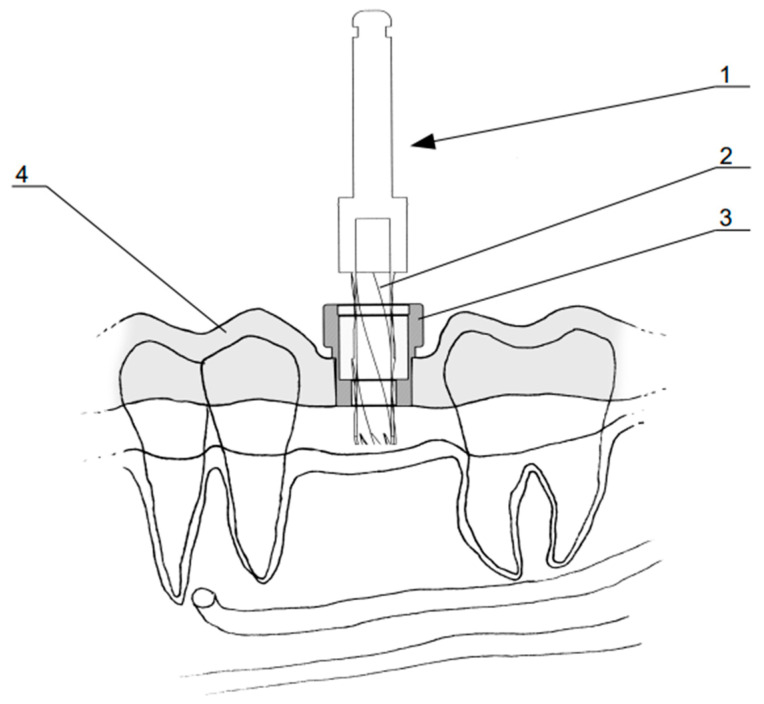
Schematic of the modified surgical guide featuring the integrated guiding sleeve and the corresponding trephine drill. 1—trephine shank, 2—trephine core, 3—trephine guiding sleeve, 4—surgical guide.

**Figure 2:**
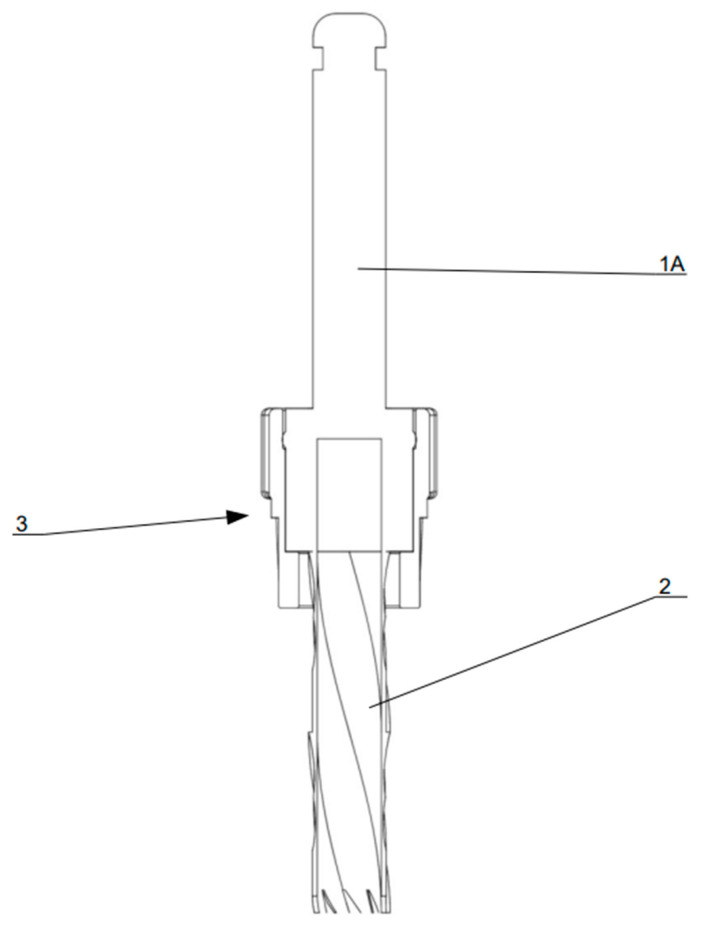
Schematic of the trephine and trephine guiding sleeve. 1A—trephine shank, 2—trephine core, 3—trephine guiding sleeve.

**Figure 3:**
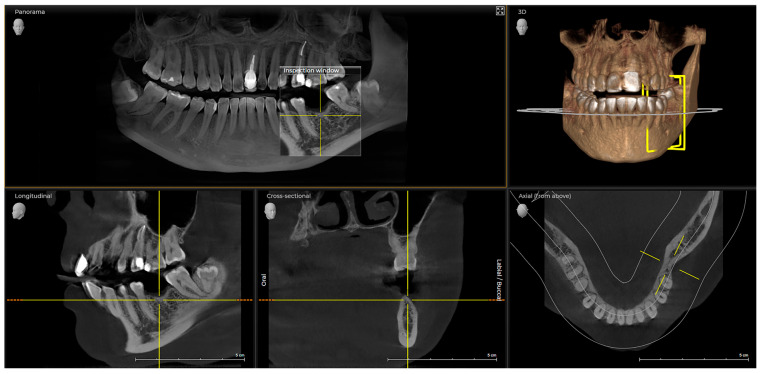
Preoperative CBCT of both arches (FOV 11 × 10 cm, voxel size 0.16 mm, 85 kV, 8 mA).

**Figure 4:**
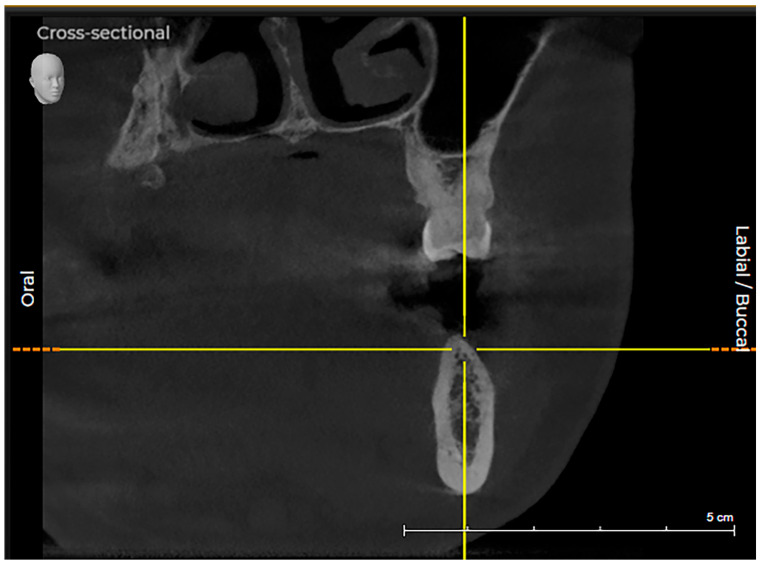
Cross-sectional CBCT view of the alveolar process in the left mandibular first molar (FDI 36) region (FOV 11 × 10 cm, voxel size 0.16 mm, 85 kV, 8 mA).

**Figure 5:**
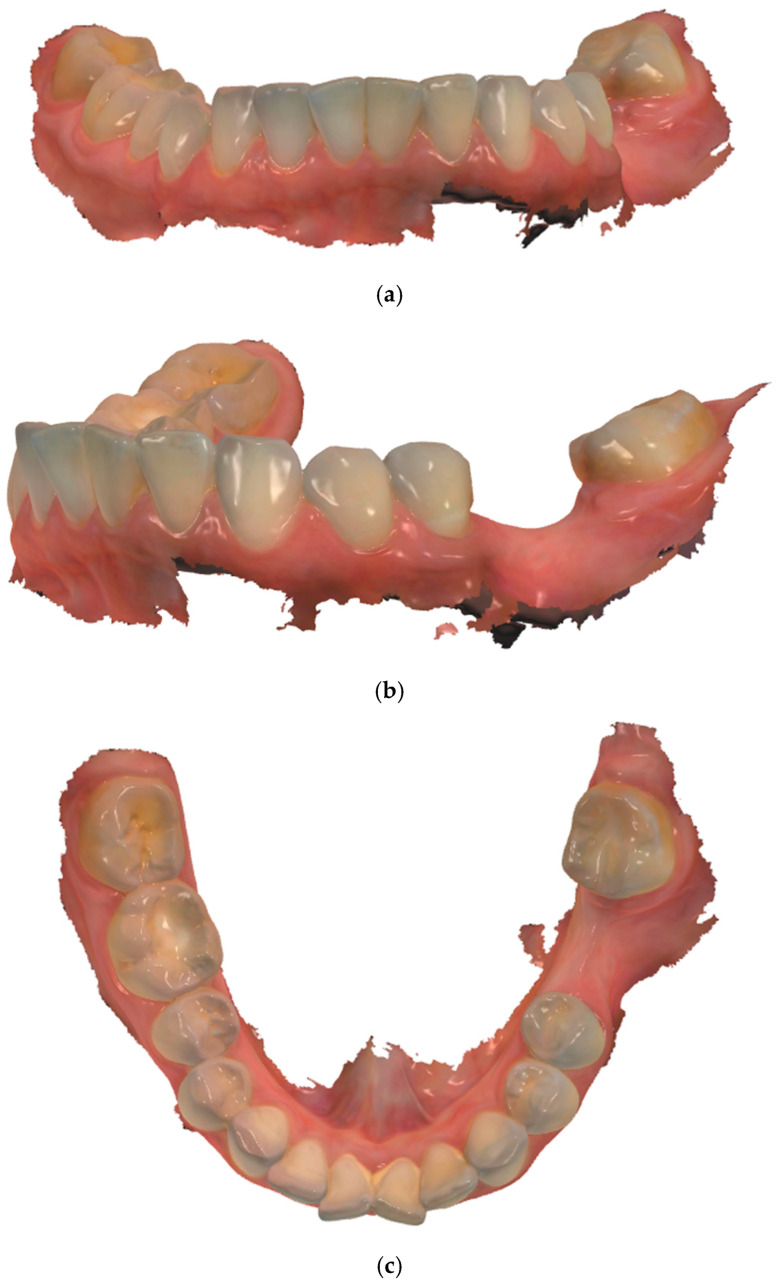
Preoperative intraoral scan of the mandibular arch. (**a**) Frontal view; (**b**) left lateral view; (**c**) occlusal view.

**Figure 6:**
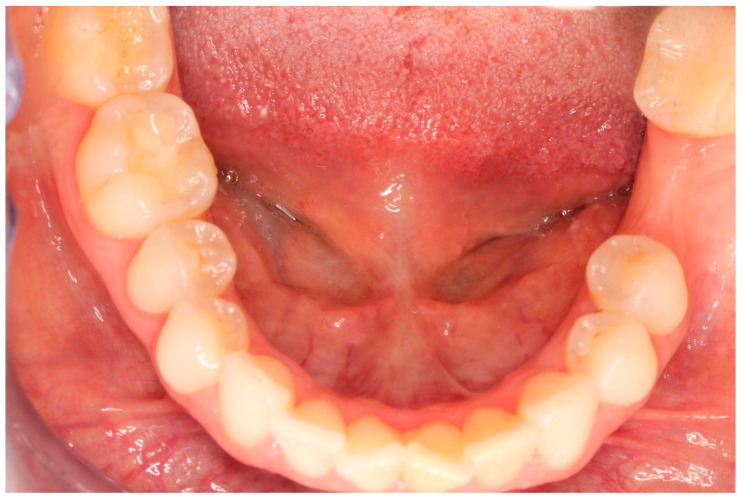
Baseline clinical condition: occlusal view of the mandibular arch.

**Figure 7:**
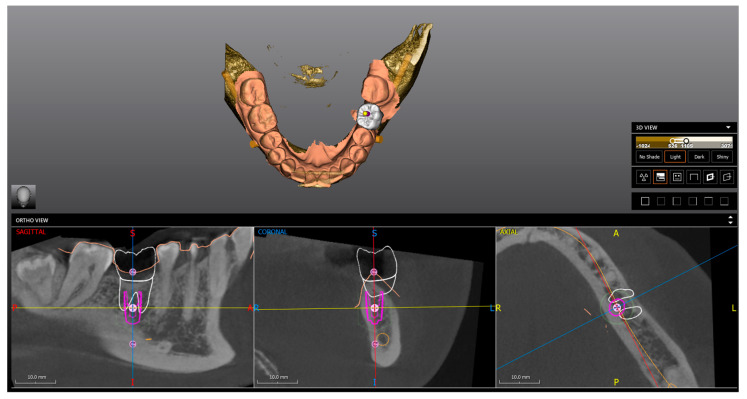
Virtual implant positioning and 3D treatment design in Zirkonzahn.Implant-Planner.

**Figure 8:**
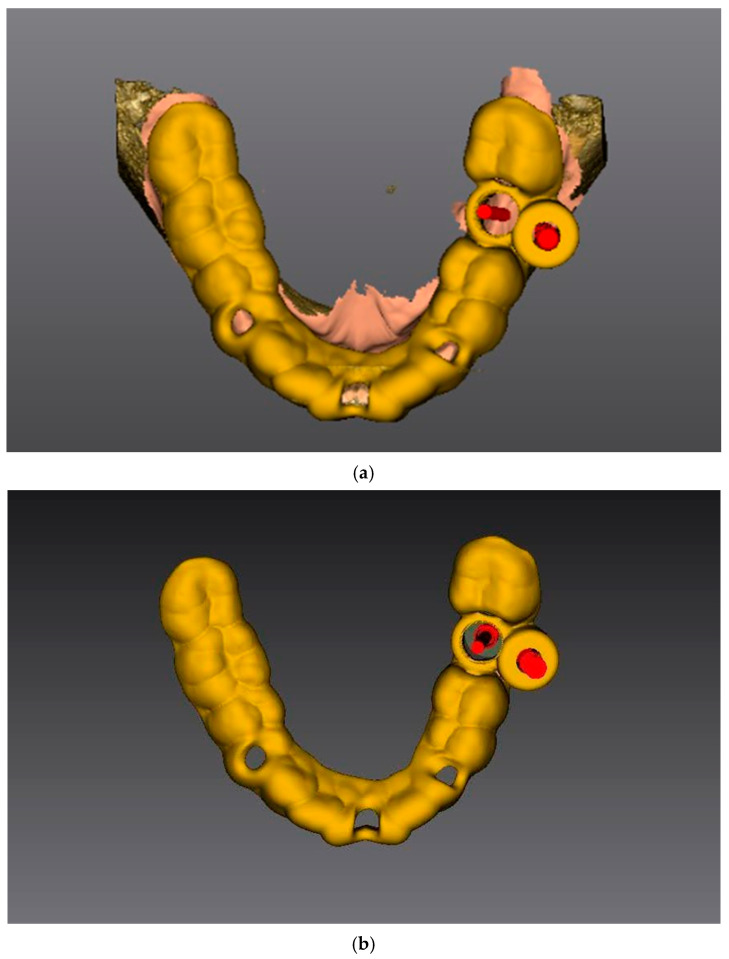

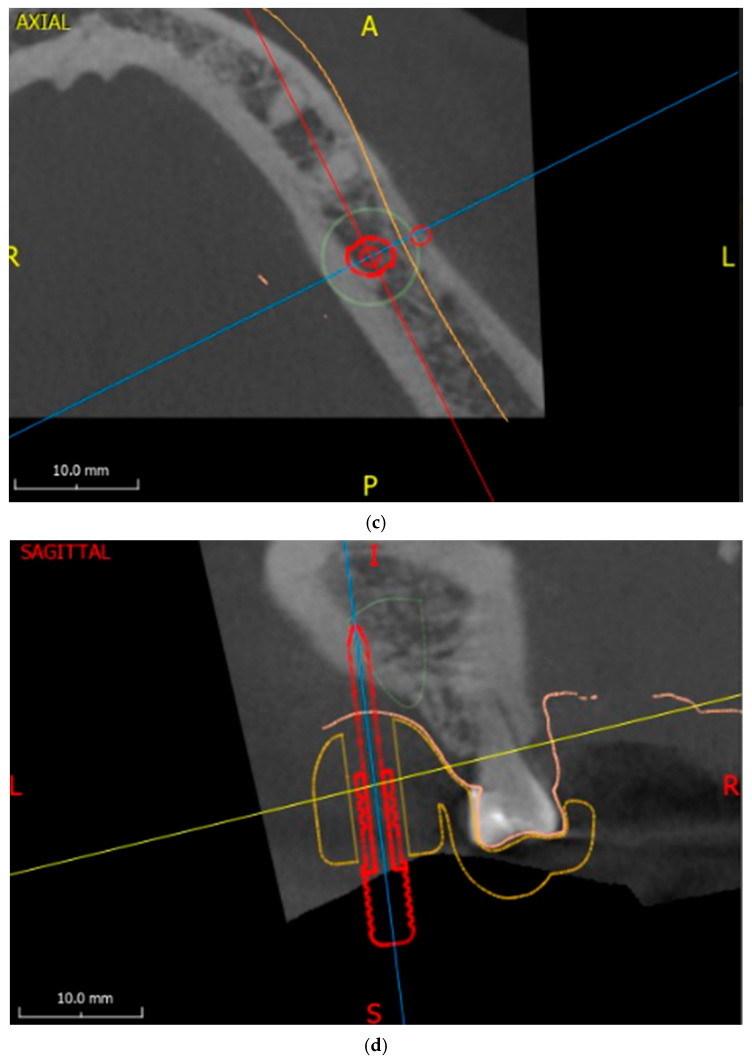

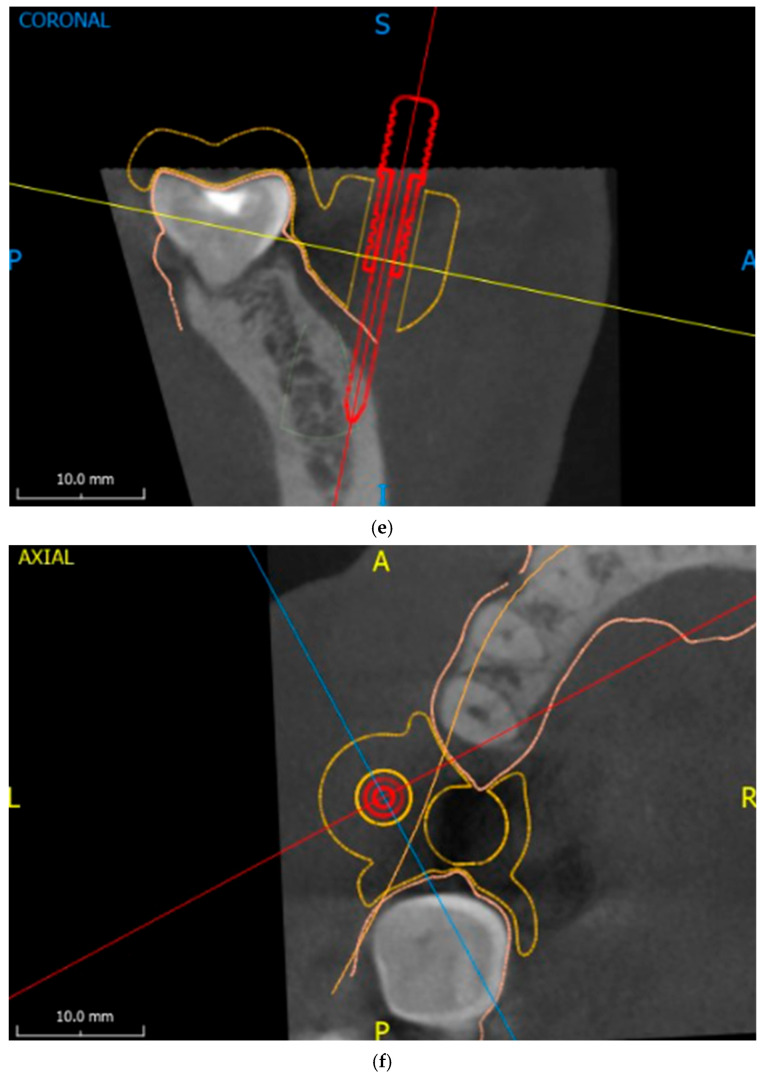
Digital design of the surgical guide. (**a**) Virtual seating of the surgical guide on the digital model; (**b**) virtual design of the surgical guide; (**c**) preoperative axial CBCT slice illustrating the spatial relationship between the implant, guide sleeve, and the pin drill; (**d**) preoperative sagittal CBCT slice illustrating the pin drill; (**e**) preoperative coronal CBCT slice illustrating the pin drill; (**f**) preoperative axial CBCT slice illustrating the pin drill.

**Figure 9:**
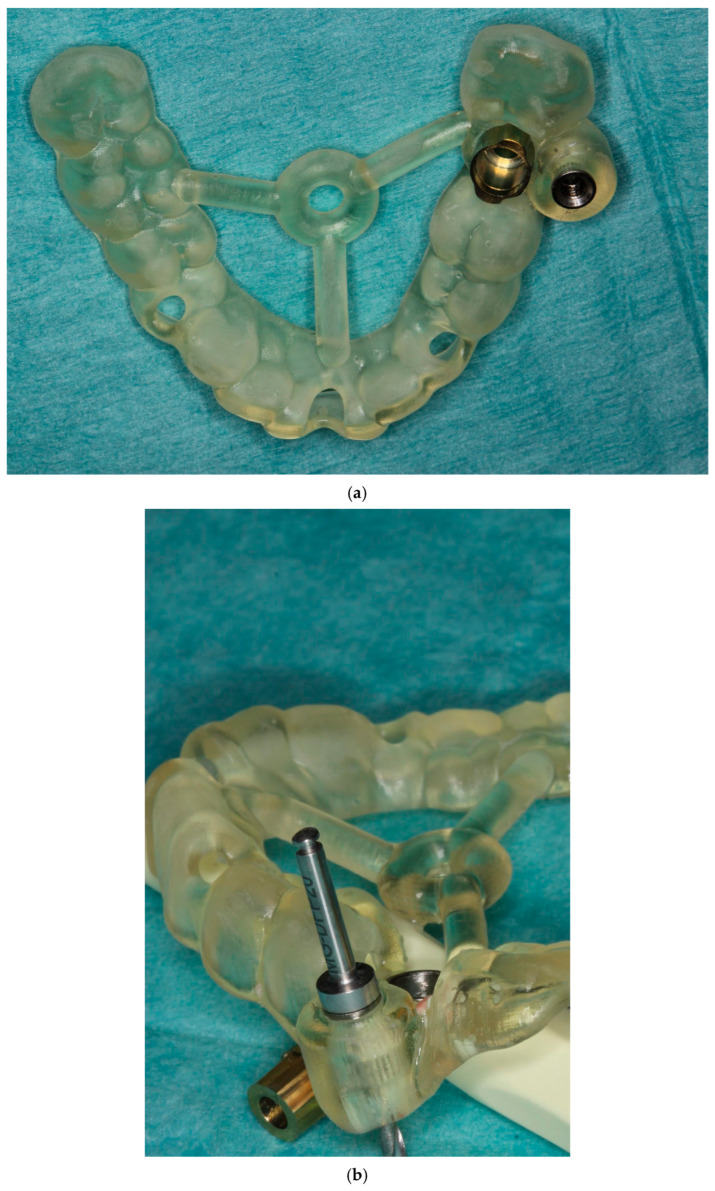
Surgical guide. (**a**) Occlusal view of the surgical guide; (**b**) lateral view of the surgical guide.

**Figure 10:**
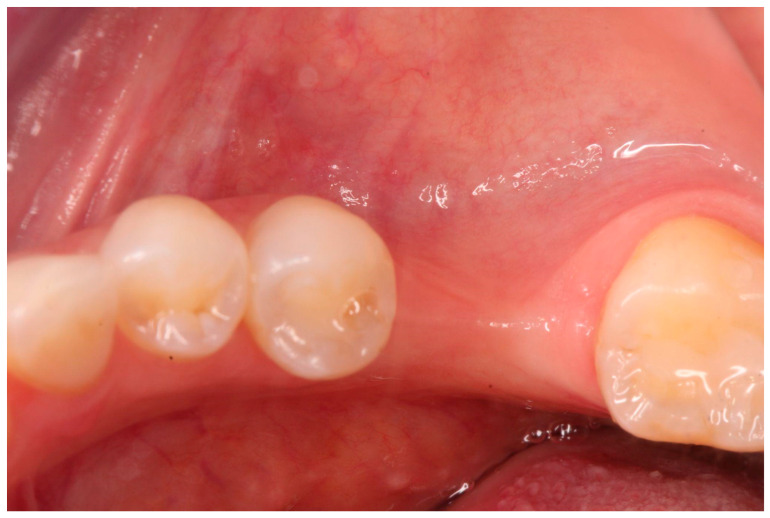
Preoperative clinical condition of the edentulous ridge at site 36.

**Figure 11:**
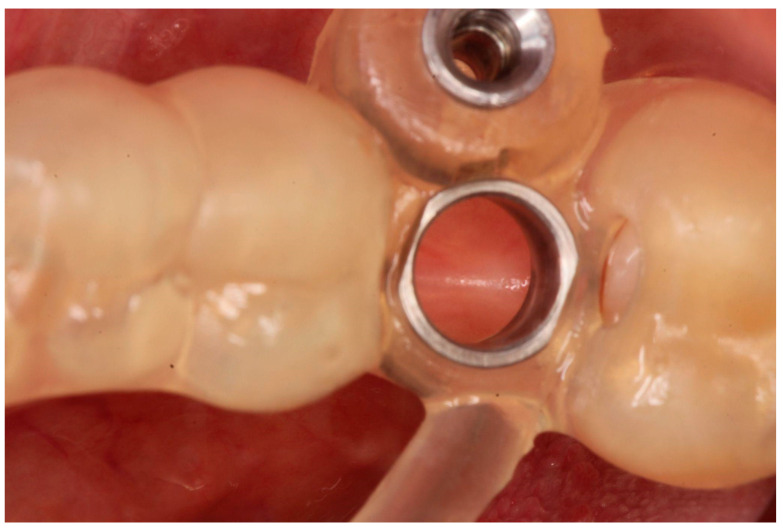
Surgical guide try-in.

**Figure 12:**
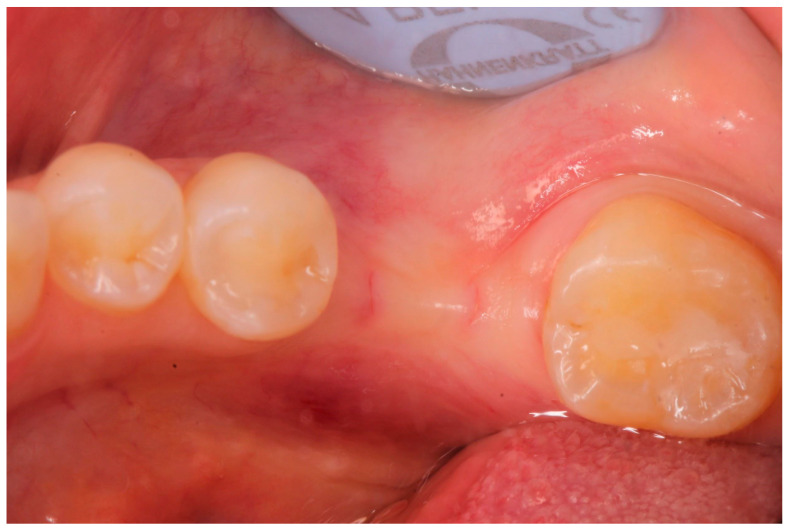
Marking the implant position.

**Figure 13:**
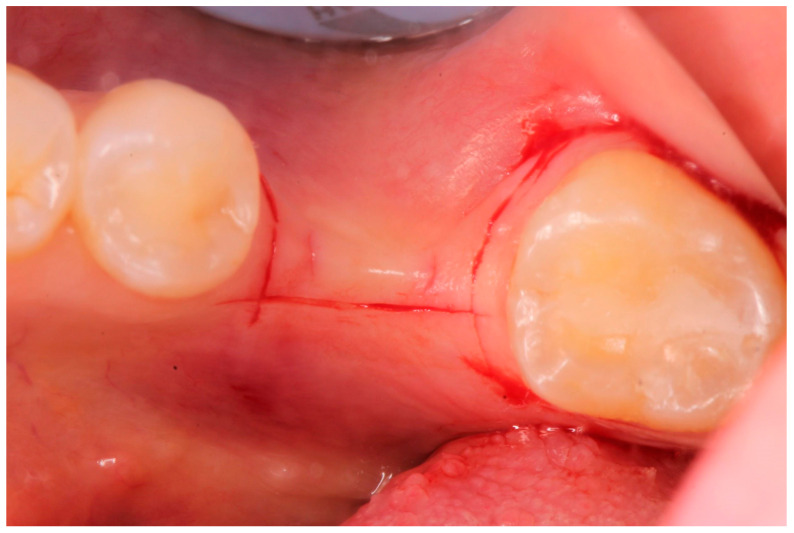
Flap incision.

**Figure 14:**
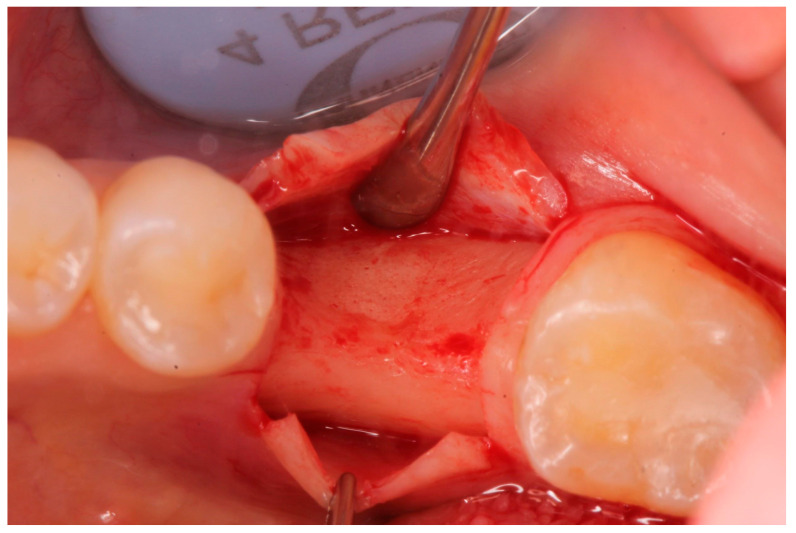
Full-thickness flap elevation.

**Figure 15:**
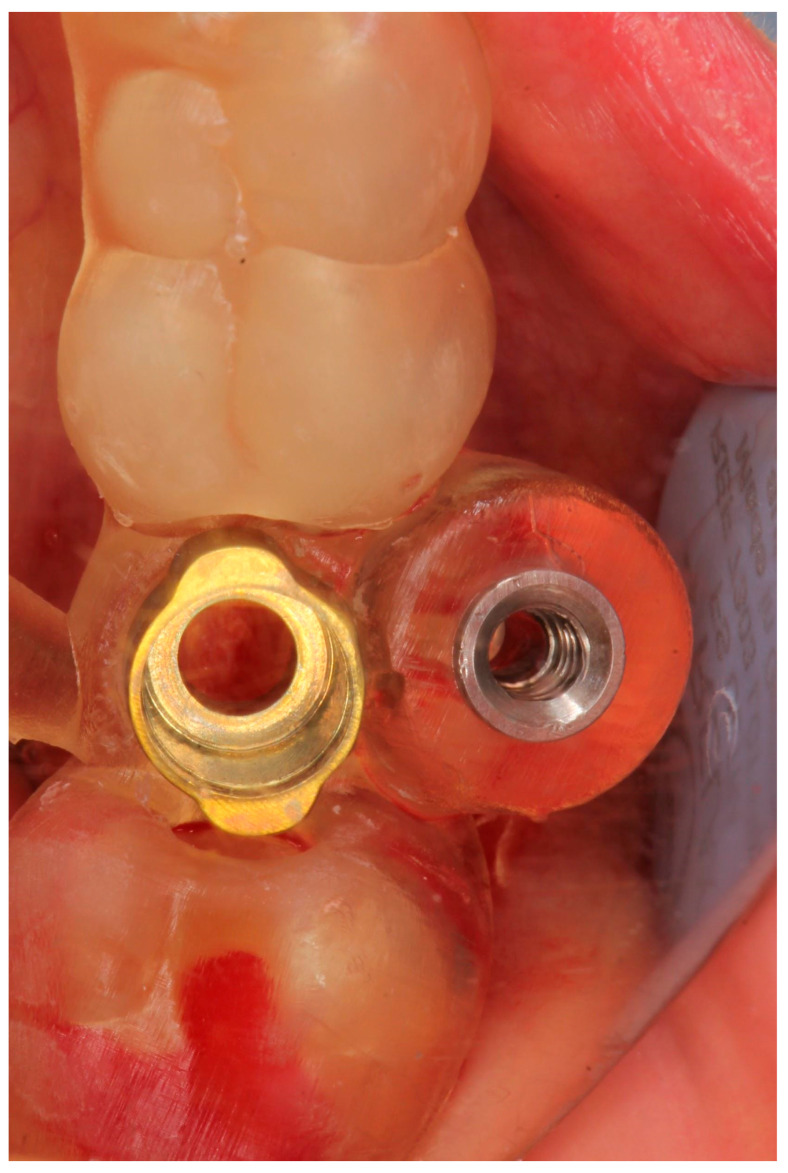
Final positioning of the 3D-printed guide following flap elevation.

**Figure 16:**
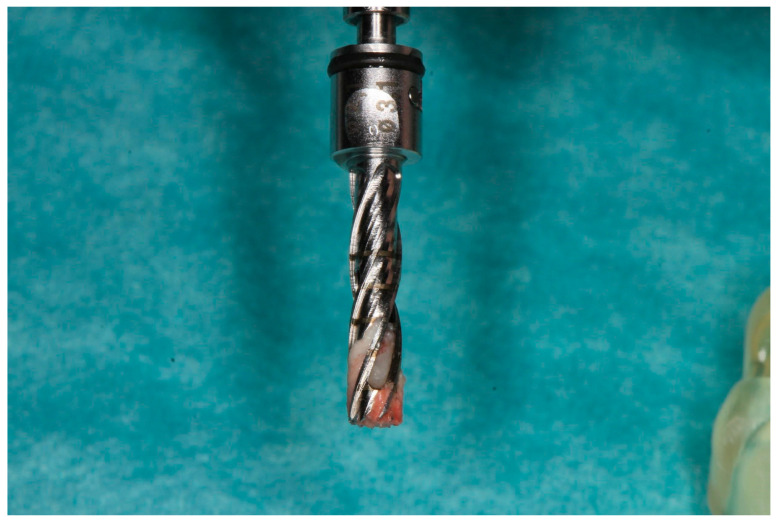
Post-osteotomy trephine with the retrieved bone cylinder.

**Figure 17:**
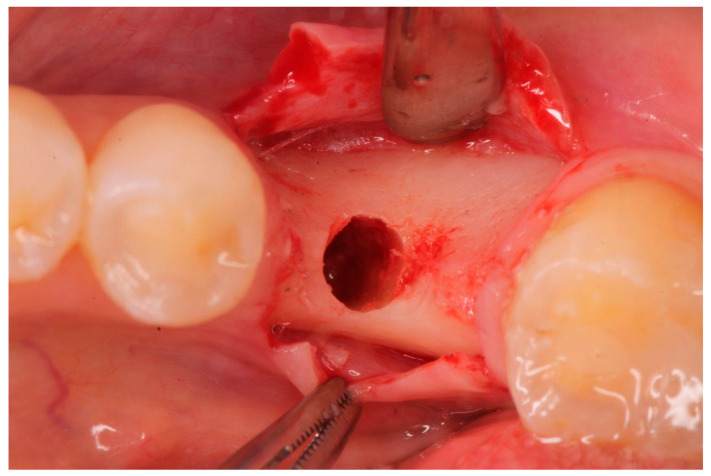
The prepared implant site at position 36.

**Figure 18:**
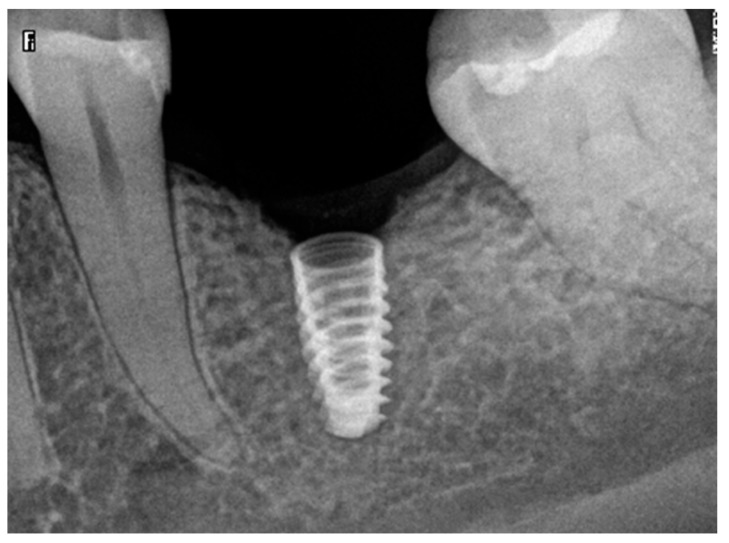
Radiographic follow-up of the implant at the left mandibular first molar (FDI 36) region.

**Figure 19:**
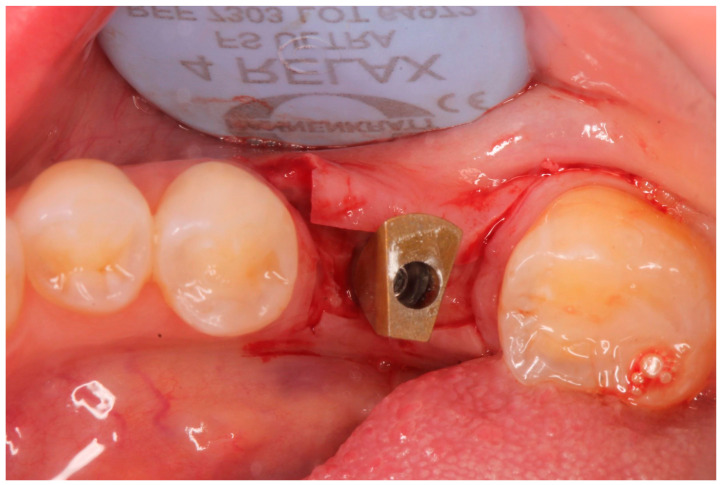
Intraoral scan of the implant at the left mandibular first molar (FDI 36) region.

**Figure 20:**
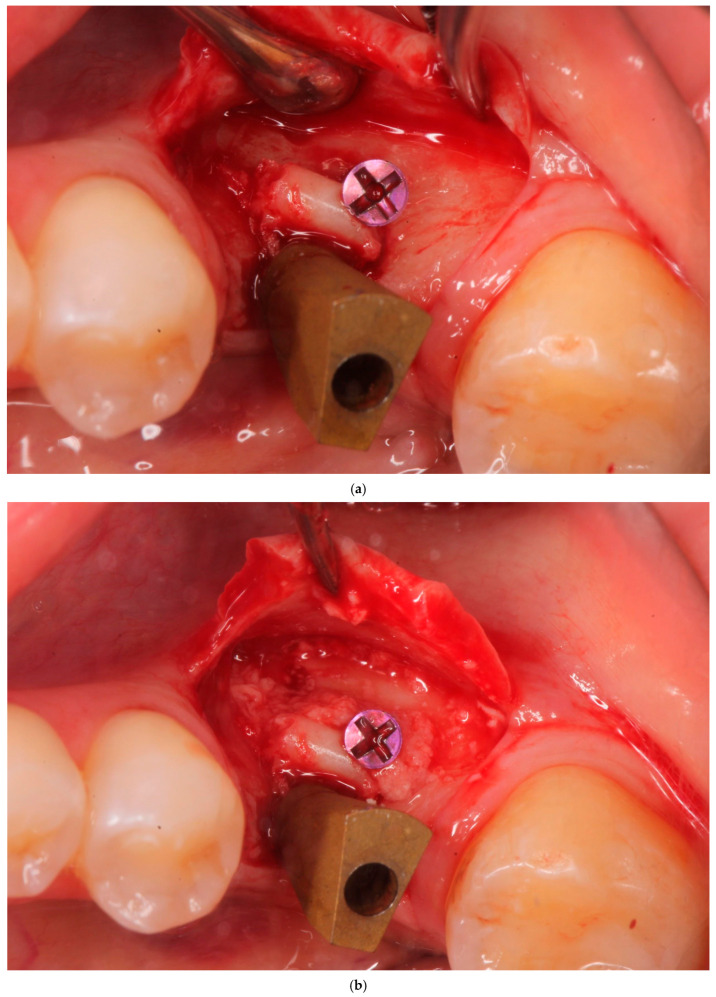
Digital CarroTrack Technique—autogenous bone augmentation and fixation screw insertion at the left mandibular first molar (FDI 36) region. (**a**) Autogenous trephine core graft with fixation screw; (**b**) final view prior to flap closure.

**Figure 21:**
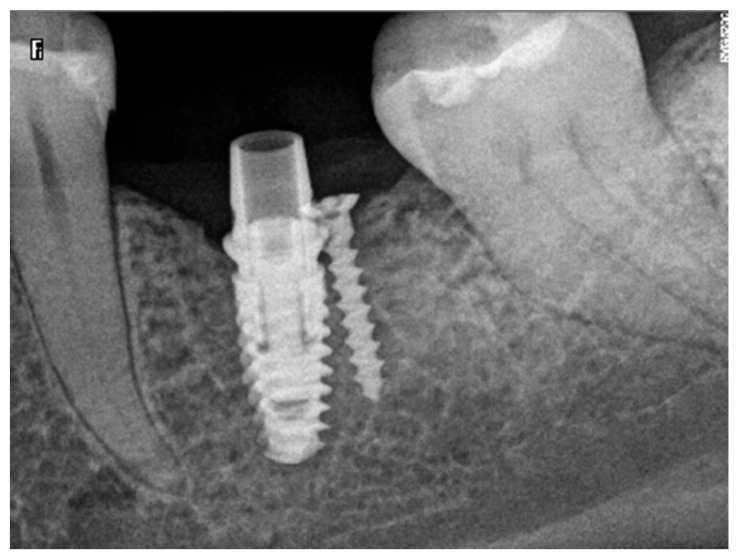
Radiographic follow-up of the implant and fixation screw at the left mandibular first molar (FDI 36) region.

**Figure 22:**
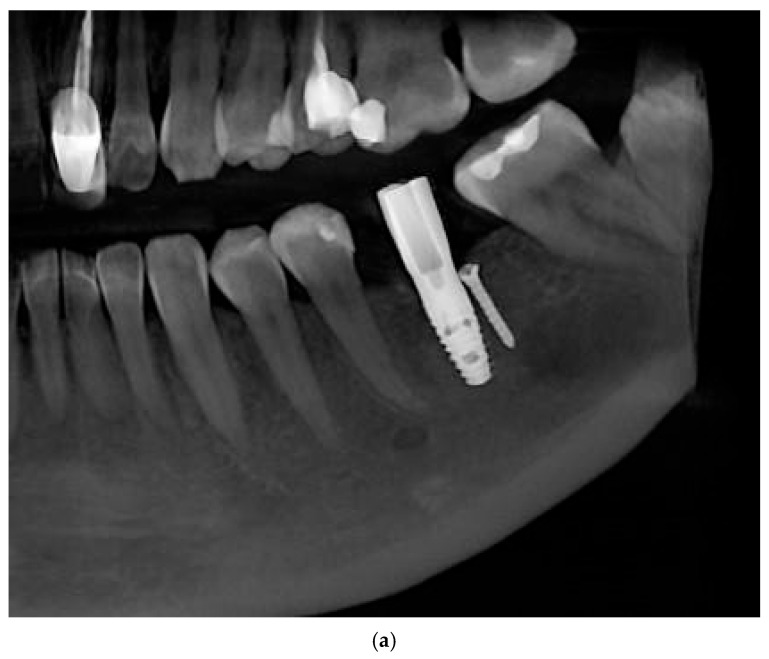

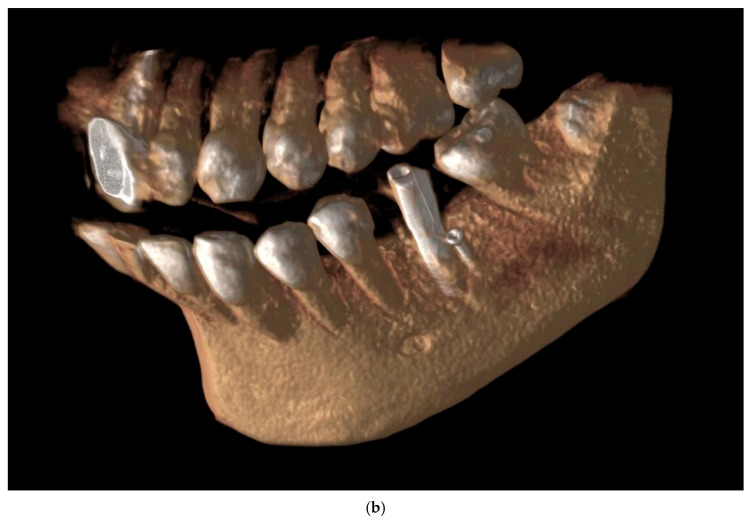
Postoperative CBCT showing the position of the implant in the left mandibular first molar (FDI 36) region (FOV 8 × 8 cm, voxel size 0.16 mm, 85 kV, 6 mA). (**a**) Panoramic view; (**b**) reconstruction.

**Figure 23:**
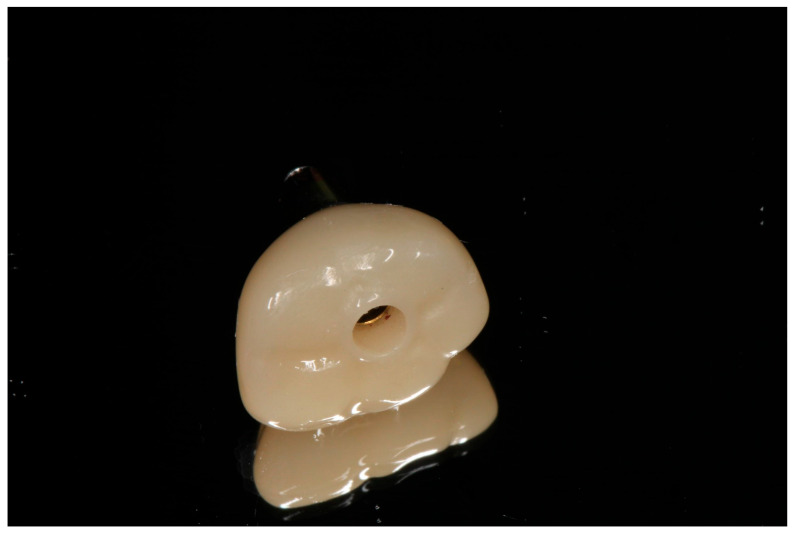
Temporary crown.

**Figure 24:**
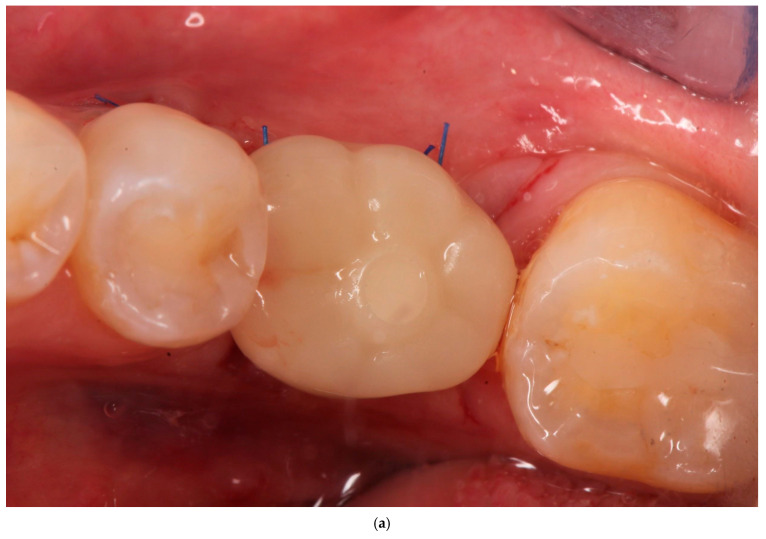

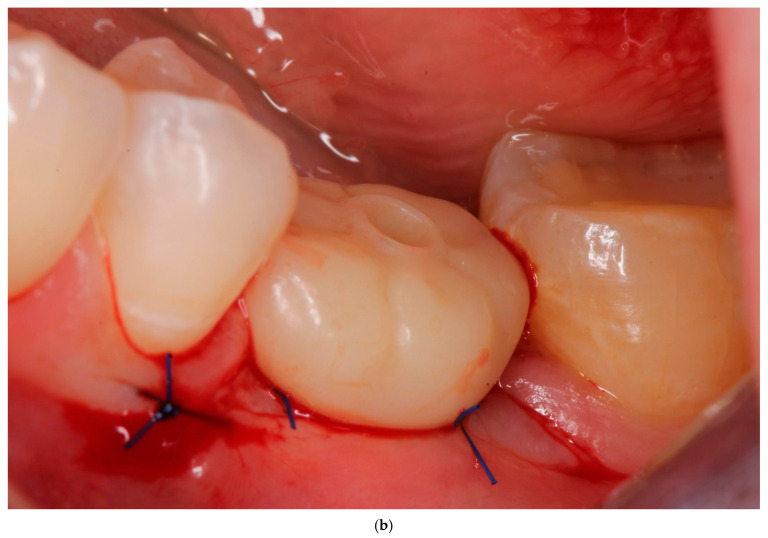
Temporary crown delivery at site 36. (**a**) Occlusal view; (**b**) buccal view.

**Figure 25:**
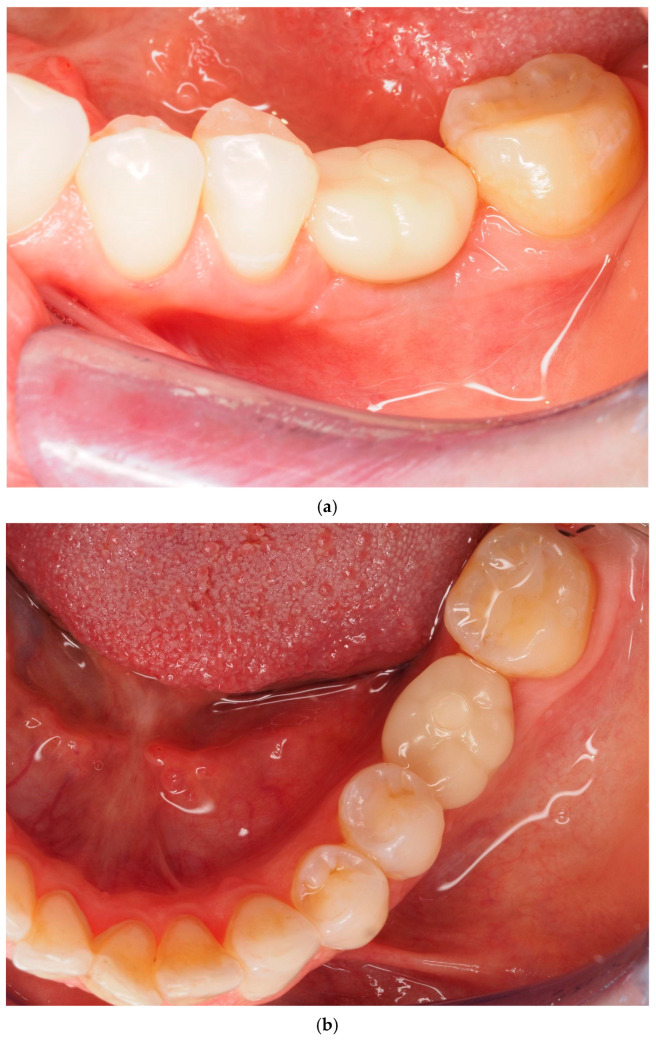
Clinical status at 7-week follow-up. (**a**) Buccal view; (**b**) occlusal view.

**Figure 26:**
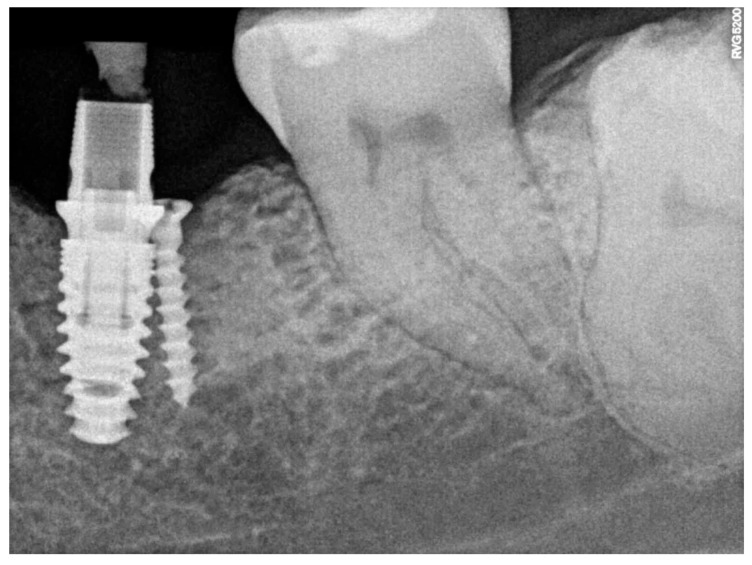
Radiographic follow-up of the implant at the left mandibular first molar (FDI 36) region, 7 weeks postoperatively.

**Figure 27:**
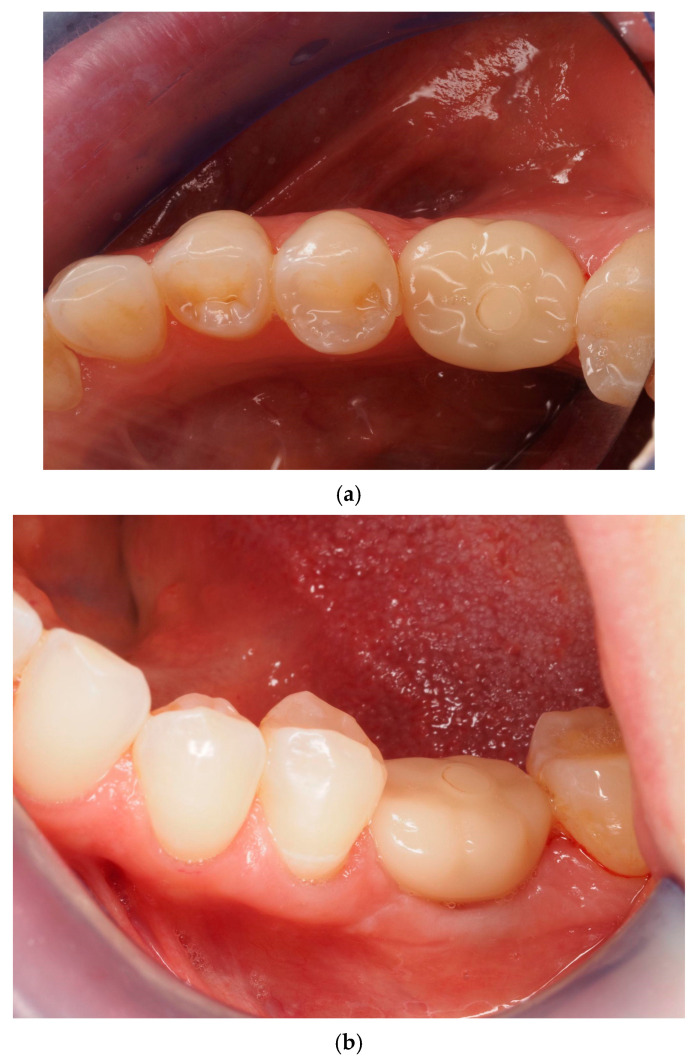
Soft tissue assessment prior to final crown delivery. (**a**) Occlusal view; (**b**) buccal view.

**Figure 28:**
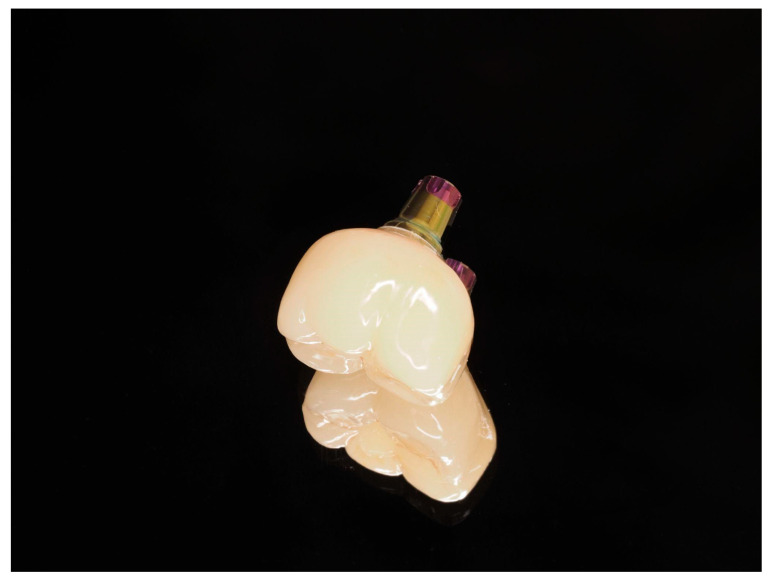
Final crown.

**Figure 29:**
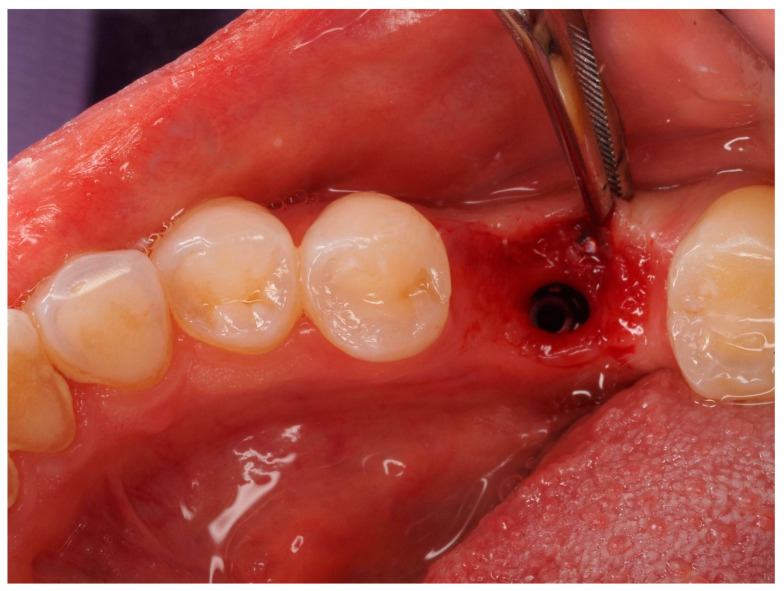
Removal of the provisional restoration and retrieval of the fixation screw.

**Figure 30:**
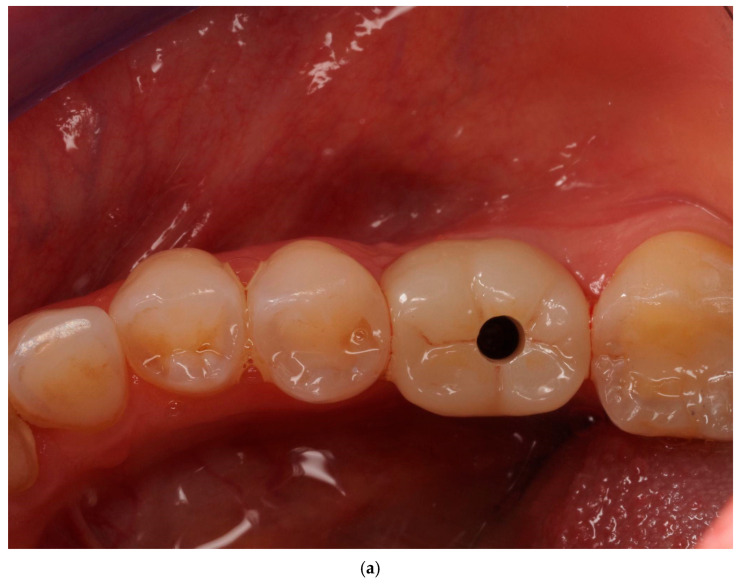

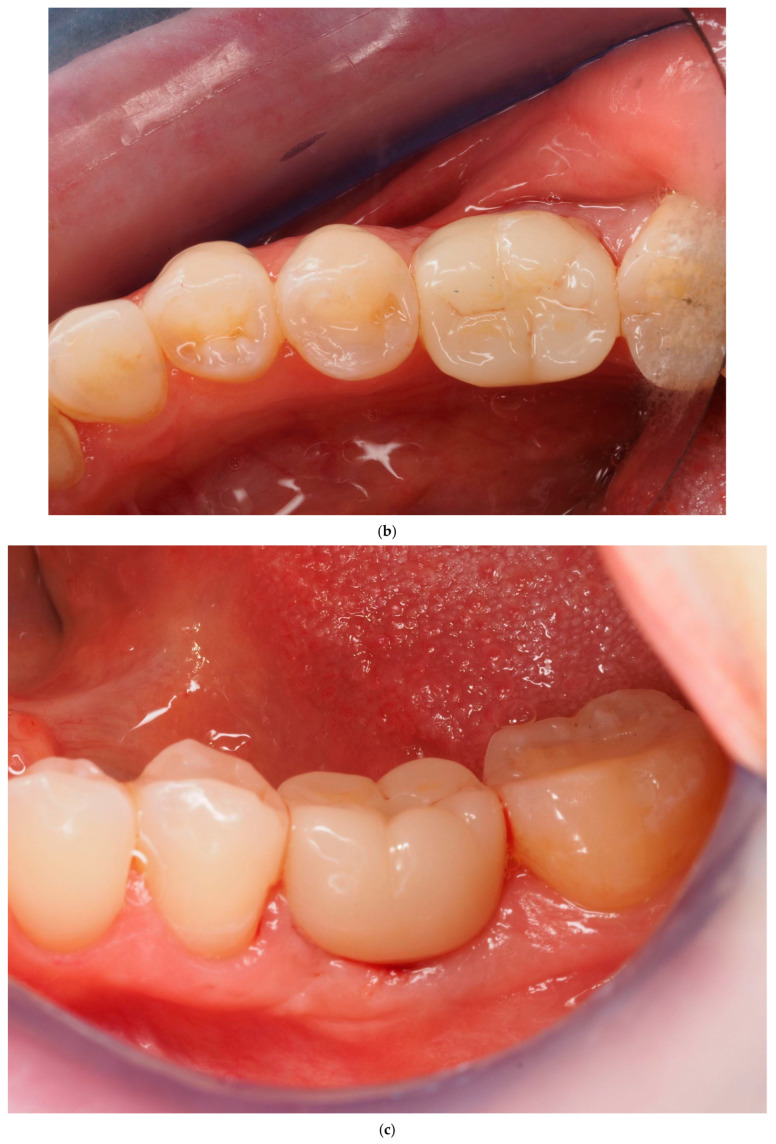
Final crown delivery. (**a**) Occlusal view before access channel closure; (**b**) occlusal view after access channel closure; (**c**) buccal view.

**Figure 31:**
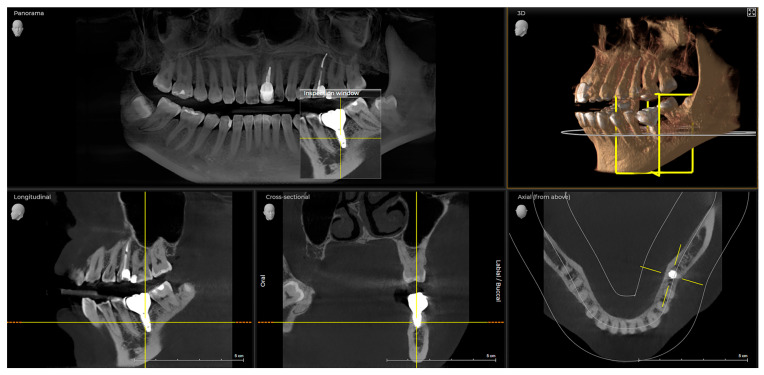
Follow-up CBCT of both arches after final crown delivery and fixation screw removal (FOV 11 × 10 cm, voxel size 0.16 mm, 85 kV, 8 mA).

**Figure 32:**
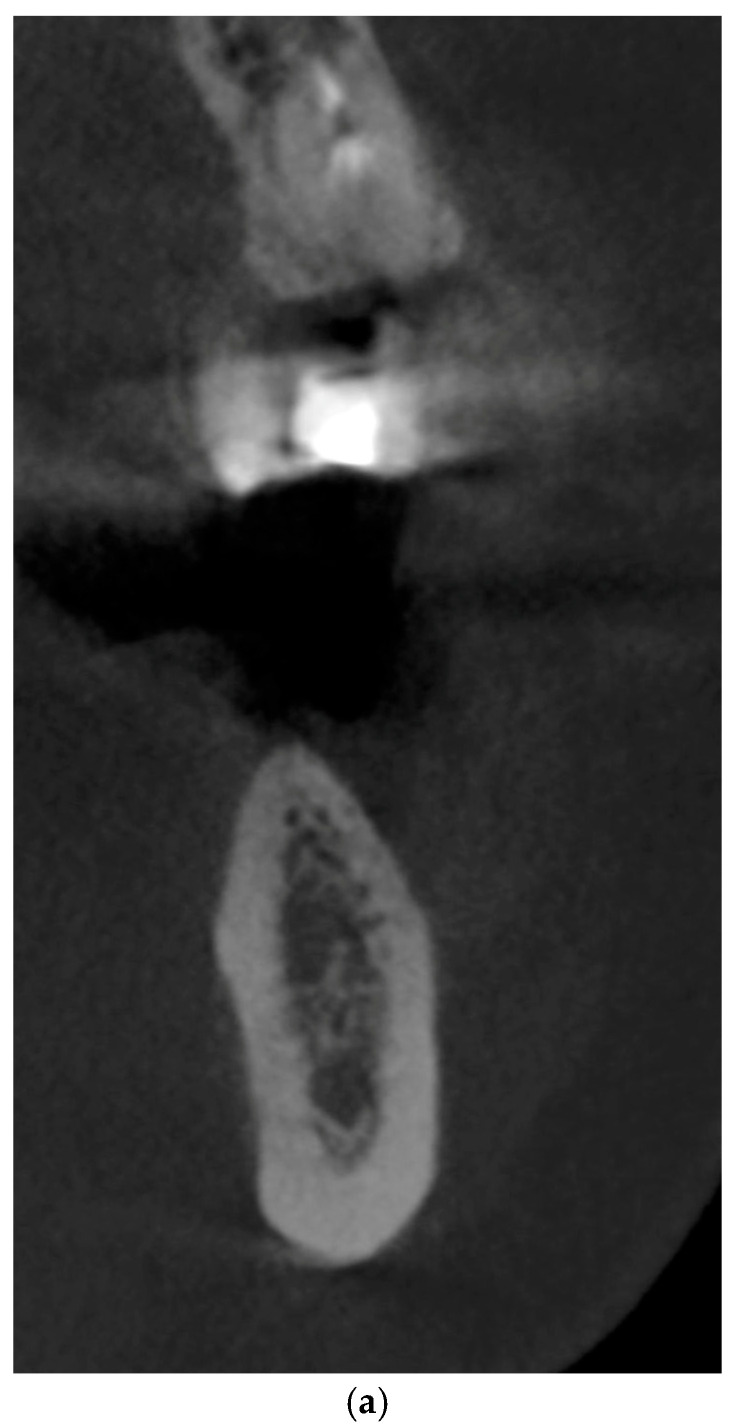

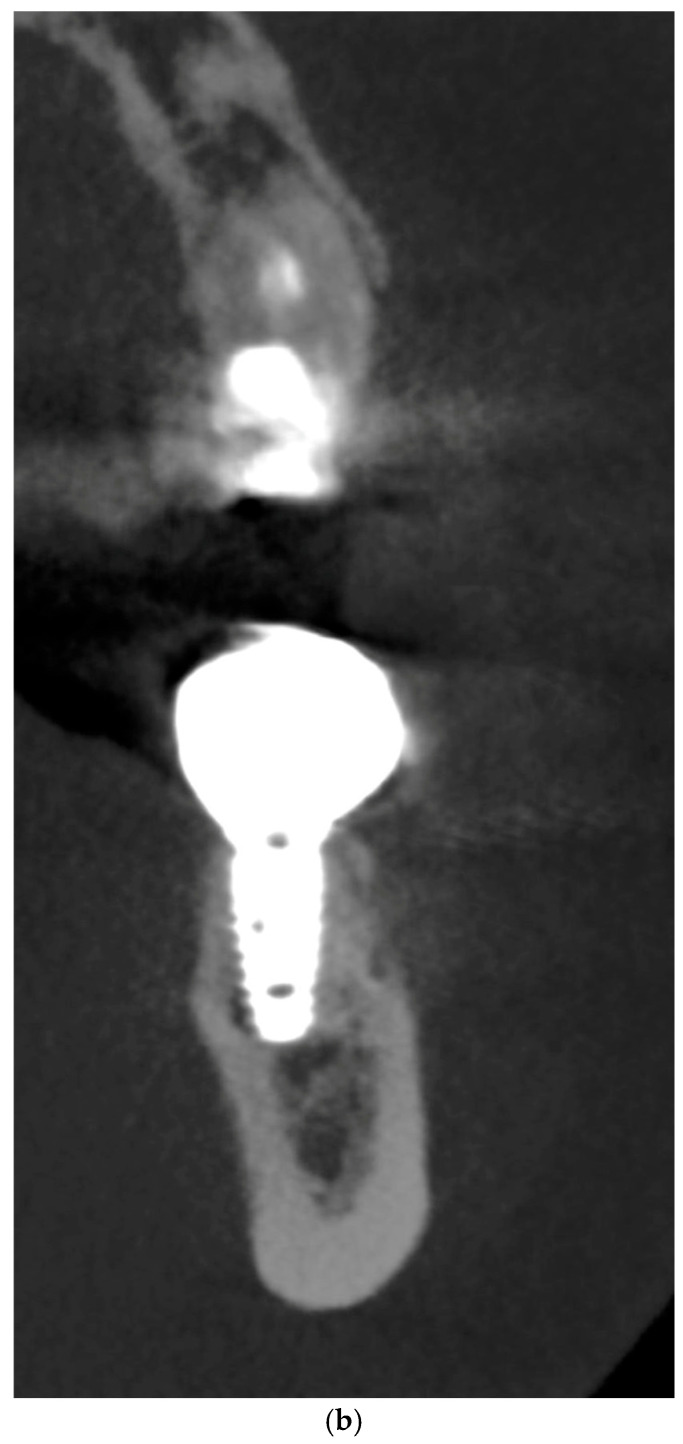

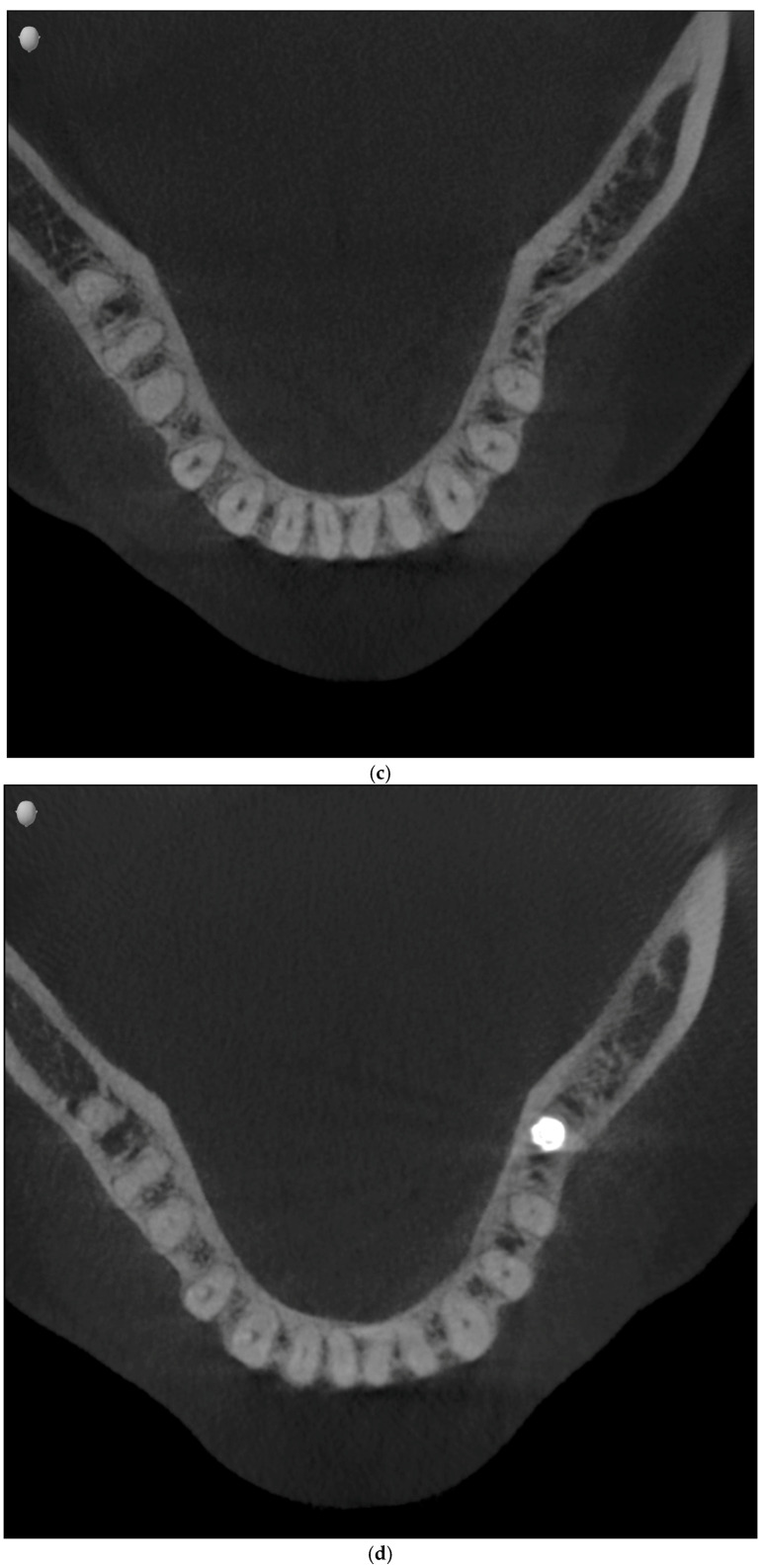
CBCT-based surgical site assessment—the left mandibular first molar (FDI 36) region. (**a**) Cross-sectional view of the left mandibular first molar (FDI 36) region before the procedure; (**b**) cross-sectional view of the left mandibular first molar (FDI 36) region with the final crown, after screw removal; (**c**) axial view of the mandible before the procedure; (**d**) axial view of the mandible after the procedure.

**Figure 33:**
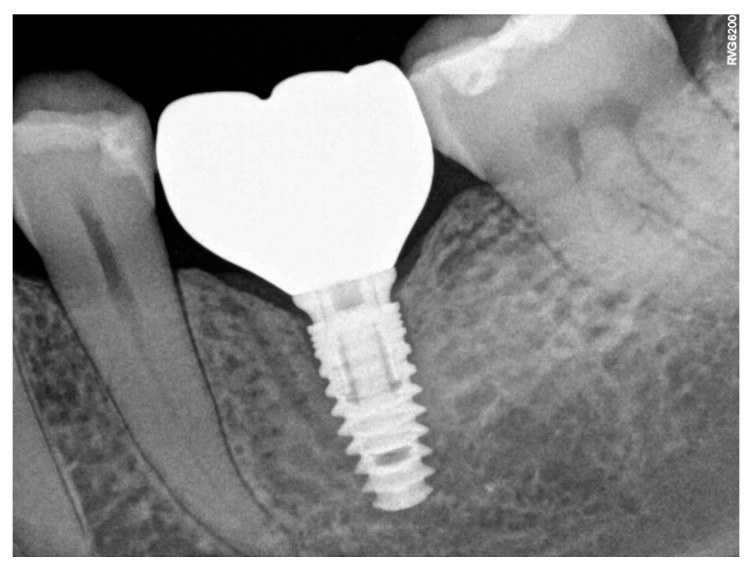
Radiographic follow-up of the implant site in the left mandibular first molar (FDI 36) region two years postoperatively.

**Figure 34:**
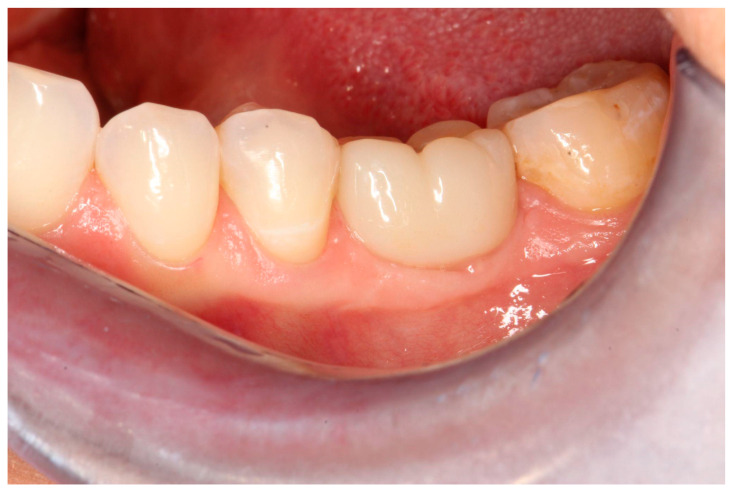
Clinical photography at 2-year follow-up: buccal view.

**Figure 35:**
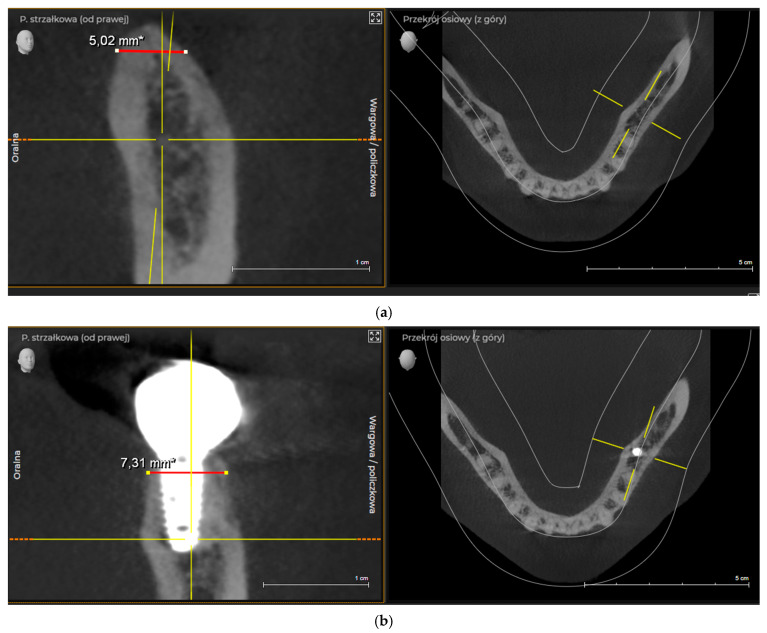
Radiographic comparison of alveolar ridge width in the left mandibular first molar region (FDI 36) before the procedure and at the 2-year follow-up. (**a**) Cross-sectional and axial view of the left mandibular first molar (FDI 36) region before the procedure; (**b**) cross-sectional and axial view of the left mandibular first molar (FDI 36) region with the final crown at the 2-year follow-up.

**Table 1:** Treatment sequence.

Treatment Stage	Description
Planning	Digital planning of the surgical guide, implant placement, fixation screw for graft stabilization, and provisional crown
Surgical protocol	Harvesting of the cylindrical bone graft, implant placement, and insertion of the fixation screw
Prosthetic protocol	Implant exposure, removal of the fixation screw and delivery of the final crown
Follow-up	Long-term clinical and radiographic evaluation

## Data Availability

The data are not publicly available due to privacy or ethical restrictions. The raw DICOM and STL files cannot be shared publicly due to patient privacy concerns and the absence of specific consent for sharing raw imaging/model datasets. Representative anonymized images and CBCT cross-sections are provided in the manuscript.
